# Research on Joint Game-Theoretic Modeling of Network Attack and Defense Under Incomplete Information

**DOI:** 10.3390/e27090892

**Published:** 2025-08-23

**Authors:** Yifan Wang, Xiaojian Liu, Xuejun Yu

**Affiliations:** Software College, Beijing University of Technology, Beijing 100124, China; wangyifansd@163.com (Y.W.); liuxj@bjut.edu.cn (X.L.)

**Keywords:** game theory, incomplete information, proximal policy optimization, multi-agent game, graph neural network

## Abstract

In the face of increasingly severe cybersecurity threats, incomplete information and environmental dynamics have become central challenges in network attack–defense scenarios. In real-world network environments, defenders often find it difficult to fully perceive attack behaviors and network states, leading to a high degree of uncertainty in the system. Traditional approaches are inadequate in dealing with the diversification of attack strategies and the dynamic evolution of network structures, making it difficult to achieve highly adaptive defense strategies and efficient multi-agent coordination. To address these challenges, this paper proposes a multi-agent network defense approach based on joint game modeling, termed JG-Defense (Joint Game-based Defense), which aims to enhance the efficiency and robustness of defense decision-making in environments characterized by incomplete information. The method integrates Bayesian game theory, graph neural networks, and a proximal policy optimization framework, and it introduces two core mechanisms. First, a Dynamic Communication Graph Neural Network (DCGNN) is used to model the dynamic network structure, improving the perception of topological changes and attack evolution trends. A multi-agent communication mechanism is incorporated within the DCGNN to enable the sharing of local observations and strategy coordination, thereby enhancing global consistency. Second, a joint game loss function is constructed to embed the game equilibrium objective into the reinforcement learning process, optimizing both the rationality and long-term benefit of agent strategies. Experimental results demonstrate that JG-Defense outperforms the Cybermonic model by 15.83% in overall defense performance. Furthermore, under the traditional PPO loss function, the DCGNN model improves defense performance by 11.81% compared to the Cybermonic model. These results verify that the proposed integrated approach achieves superior global strategy coordination in dynamic attack–defense scenarios with incomplete information.

## 1. Introduction

With the rapid development of information technology, networks have become an indispensable part of modern social, economic, political, and cultural activities. However, while the widespread adoption of networks significantly improves the efficiency of information flow, it also introduces unprecedented security challenges. Traditional network defense systems typically rely on centralized decision-making models, in which a single decision unit oversees and controls the entire network. However, as attack methods become increasingly sophisticated and intelligent, centralized defense systems face unprecedented challenges in adapting to rapidly changing network environments.

In traditional network defense systems, centralized defense models are often employed, where a single decision unit performs global observation and judgment over the entire network. However, as attack techniques continue to evolve, a single defense strategy can no longer cope with increasingly complex security threats. Defense systems are often limited to partial observations of the network state, resulting in delayed or incorrect decisions and thus failing to provide effective protection.

Moreover, as the scale of the network expands, the volume of information that centralized systems must process grows exponentially, leading to processing bottlenecks and constrained emergency response capabilities. In the current field of cybersecurity, attack and defense problems are increasingly abstracted as strategic adversarial processes within a game-theoretic framework. However, unlike complete-information games in classical game theory, real-world network attack and defense scenarios are inherently games of incomplete information. This is because, on one hand, defenders are typically unable to observe the full state of attackers—including their intent, resources, potential methods, and concrete action plans—since such critical information is often concealed within complex traffic and obfuscation techniques. On the other hand, attackers also find it difficult to fully understand the real-time deployments, detection capabilities, and response mechanisms of the defenders, relying instead on reconnaissance, probing, and gradual infiltration to infer defense strategies. This partial observability compels both sides to base their decisions on probabilistic inference, belief updating, and strategic reasoning rather than deterministic information.

Moreover, modern network defense environments inherently exhibit multi-agent characteristics. In large-scale distributed networks, defense tasks often involve multiple autonomous decision units, each possessing independent observation perspectives and local control capabilities. They must collaborate under limited local information, sharing data, coordinating strategies, and conducting distributed optimization to achieve global defense objectives. Meanwhile, attackers often employ multi-point collaborative, multi-stage distributed attack strategies—such as botnets, distributed denial-of-service attacks, and advanced persistent threats—all of which exhibit distinct multi-agent characteristics in terms of attack surface and tactical deployment. Therefore, adopting multi-agent strategies on the defense side is both necessary and imperative for resisting such attacks and integrating broader situational awareness. Hence, the overall network attack–defense landscape is not only a typical game of incomplete information but also a joint game among multiple agents, where each agent’s local actions directly influence the system-wide defense effectiveness and the equilibrium of the game.

Game theory has been widely introduced to model the strategic interactions and dynamic evolution between attackers and defenders. Such studies provide defenders with theoretical frameworks for strategy optimization, enabling them to maximize their utility in complex games, yet providing effective game modeling under dynamic attack–defense environments remains a major challenge. In real-world applications, ensuring efficient strategy coordination among agents under incomplete information—while avoiding local optima or conflicts—remains an open and critical problem. Furthermore, graph neural networks [1,2] have recently been employed to extract complex relational and latent threat patterns from network topologies, yet most existing work focuses on static or quasi-static graphs, with limited modeling of temporal dynamics, attack evolution, and multi-stage interactions in dynamic environments. Moreover, game theory, multi-agent systems, and GNNs are often treated as separate technologies, lacking an integrated framework capable of delivering end-to-end and highly robust system-level defense solutions.

To address the above challenges, this paper proposes a multi-agent network defense approach based on joint game-theoretic modeling named JG-Defense (Joint Game-based Defense). This approach exhibits two significant advantages: (i) A strategy optimization mechanism that integrates game theory with the Proximal Policy Optimization framework. It enables dynamic adjustment of agent strategies through a Bayesian belief [3]-updating mechanism and introduces a joint game-theoretic loss function to effectively enhance the rationality and strategic behavior of agents under incomplete information. (ii) An integrated dynamic defense architecture that combines a multi-agent communication system with a Dynamic Communication Graph Neural Network. By embedding dynamic GNNs into the agent models, the model enhances its ability to capture node importance, attack path evolution, and topological changes in dynamic networks, thereby improving the perception and response to complex attacks [4]. A multi-agent communication mechanism is also introduced into DCGNN (Dynamic Communication Graph Neural Network), enabling agents to dynamically exchange local observations based on graph adjacency, thus achieving effective strategy coordination and joint optimization, which improves both the system’s overall responsiveness and the consistency and robustness of global strategies.

The remainder of this paper is organized as follows. Section 2 reviews and analyzes related work, focusing on network attack–defense modeling under incomplete information, graph neural networks, and multi-agent games. Section 3 presents the overall architecture and design flow. Section 4 details the construction and optimization of individual agent loss functions, particularly how Bayesian belief updating is integrated into the reinforcement learning framework. Section 5 explores the roles of multi-agent systems and GNNs in complex attack–defense scenarios, including their applications in feature extraction, strategy coordination, and information sharing. Section 6 presents experimental setups and results, providing comprehensive validation of the proposed algorithm’s effectiveness, robustness, and practical advantages.

## 2. Related Work

### 2.1. Modeling of Incomplete Information in Network Environments

The core focus of current research on network attack–defense games lies in optimizing adversarial strategies under complex conditions by constructing game-theoretic models to capture the dynamic interactions between attackers and defenders. These studies span multiple layers of defense mechanisms, including resource optimization, threat detection, and deception-based techniques. Driven by advancements in machine learning and big data, defensive strategies have gradually evolved toward data-driven adaptive decision-making, yielding notable improvements in threat prediction and response efficiency. However, many existing studies still face considerable challenges in practical deployment, primarily due to overly simplified modeling and reliance on the assumption of perfect information.

For instance, Zhang Hengwei [5] proposed an epidemic-based attack–defense model that utilizes differential games to describe strategic interactions. While this model effectively abstracts key characteristics of adversarial behavior and simplifies the defense process to some extent, its abstraction of complex strategies and reliance on perfect information limit its realism and applicability in actual network scenarios. In practice, both attackers and defenders rarely possess complete knowledge of each other’s internal states, which further restricts the model’s effectiveness in real-world applications.

To address the limitations of perfect information games, Amrita Dahiya [6] integrated economic theories to propose an incentive mechanism based on Bayesian games and reputation scoring, aimed at mitigating DDoS attacks [7] and strengthening network defense. This mechanism effectively handles the uncertainty of malicious users, promotes legitimate behavior within the network, and suppresses potential malicious activities, thereby demonstrating greater flexibility and adaptability in countering DDoS threats.

Moirangthem Tiken Singh [8] introduced an online learning model that assists defense agents in learning attacker behaviors through interaction. The model is based on a stochastic game with bounded rationality and avoids the problem of state explosion by solving stateless stochastic games. In a related study, Zhang et al. [9] proposed a method to periodically collect vulnerability information in the network and modeled attack–defense behaviors based on the lifecycle of vulnerabilities, allowing them to quantify the security posture.

In recent years, the rapid development of deep reinforcement learning has further propelled the application of game theory in network security. For example, Ebrahimi et al. [10] applied adversarial reinforcement learning methods to significantly enhance the robustness of three types of malware detectors against adversarial variants, achieving improvements by factors of 4×, 7×, and 11×, respectively. Yutao et al. [11] utilized the DQN method to detect botnet traffic, greatly improving model adaptability across multiple datasets. Additionally, Yali Wu et al. [12] combined DQN with bidirectional long short-term memory networks to successfully detect zero-day attacks [13], demonstrating stronger generalizability and broader applicability.

### 2.2. Applications of Graph Neural Networks in Network Environments

With the rapid advancement of graph neural network (GNN) technologies, their applications in network security have attracted increasing attention. GNNs [14,15] are capable of efficiently modeling complex graph-structured data and performing dynamic analysis and prediction of malicious behaviors through information propagation and aggregation across nodes.

First, the use of GNNs in intrusion detection systems has been extensively explored. Studies have demonstrated that GNNs can effectively capture intricate relationships among network nodes, thereby enabling the identification of potential intrusions. For example, Zhong et al. [16] conducted a comprehensive survey on the application of GNNs in IDS, summarizing current challenges and future directions. They noted that GNNs have achieved significant progress in detecting abnormal network traffic. Meanwhile, Wang et al. [17] proposed a graph perturbation defense method specifically designed to resist membership inference attacks against GNN models, further enhancing their robustness in cybersecurity contexts.

In addition, Takiddin et al. [18] investigated the use of GNNs for detecting false data injection attacks and introduced a generalized GNN framework. Their research demonstrated the effectiveness of GNNs in large-scale dynamic systems, showing that GNNs can exploit node dependencies to identify forged attack data. Similarly, Duan et al. [19] proposed a dynamic line graph-based intrusion detection method that incorporates semi-supervised learning techniques to improve recognition of intrusion behaviors in evolving network environments. Their approach dynamically adjusts graph structures to adapt to changing network conditions, effectively addressing the limitations of traditional methods in terms of timeliness and flexibility.

### 2.3. Current Research on Multi-Agent Network Games

In network attack–defense games, the involvement of multiple agents is essential. Current research in multi-agent network games mainly focuses on the design of multi-agent game models, the application of multi-agent reinforcement learning (MARL), collaborative defense strategies, and the generation of adversarial examples. Models based on zero-sum games, Markov decision processes [20], and generative adversarial networks [21] are widely applied, significantly enhancing the adaptability of defense strategies in dynamic environments.

Yunlong Tang [22] employed a hierarchical MARL approach as the driving force behind game evolution, solving for Nash equilibria sequentially and forming autonomous dynamic defense strategies. This method achieved high scores in the CASE2 experimental setting. Shifei Ding [23] leveraged GNNs to efficiently extract and utilize rich information from neighboring agents within graph structures, enabling the generation of high-quality and expressive feature representations for cooperative tasks. Miaojiang Chen [24] proposed a game-theoretic deep reinforcement learning framework based on post-decision states, designed to intelligently defend against strategic adversaries. Tao Li [25] summarized that multi-agent learning in network games can be categorized into three aspects: agents’ observation capabilities, agents’ beliefs about others, and strategy generation based on these beliefs. The research presented in this paper also builds upon these three core components.

## 3. Technical Architecture and Overall Framework Design

**Figure 1:**
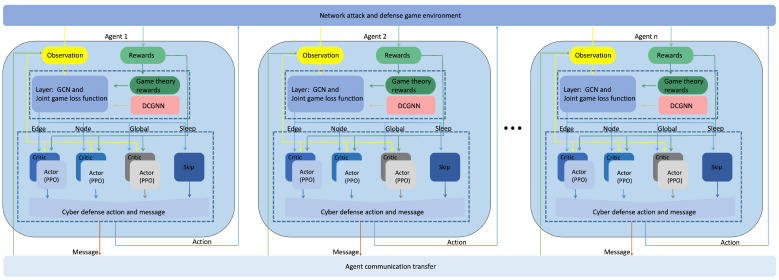
Overall technical architecture of the JG-Defense method.

In complex and dynamic network attack–defense environments, implementing an efficient and collaborative multi-agent defense strategy requires a system-level architecture that supports information sharing, strategic coordination, and game-aware optimization. To address this, we propose the technical architecture illustrated in Figure 1, where the overall design integrates a multi-agent communication network, a dynamic graph neural network, and a game-theoretic reinforcement learning optimization framework, forming a modular and integrated Joint Game-based Defense system—JG-Defense.

This approach introduces a network defense optimization framework—JG-Defense —that integrates game theory, multi-agent reinforcement learning, and graph neural networks. Specifically, game theory provides the theoretical foundation for strategic interaction and utility optimization both among defender agents and between defenders and attackers. Within the JG-Defense framework, defender agents are explicitly involved in the game process: on one hand, defender agents directly engage in adversarial games against attacker agents, optimizing their strategies to resist intrusions and disruptions; on the other hand, defender agents collaborate via the communication mechanisms output by the DCGNN, enabling real-time sharing of local observations and strategic intent, thus creating synergy in game-theoretic decision-making. In particular, the DCGNN not only is responsible for feature extraction of dynamic network topology and attack evolution patterns but also serves as a communication hub for defender agents, allowing each agent to achieve close collaboration by sharing real-time information and strategic recommendations. Furthermore, through the joint game-theoretic loss function (corresponding to the “game theory rewards” in Figure 1), the relationships between the strategies of defender agents in the game process are made explicit, encouraging each agent to consider not only its own immediate payoff but also the strategies of others, thereby enhancing the overall coordination and game-theoretic utility of the defense strategy.

At the base layer of the architecture is the multi-agent communication network, which forms the foundation for system-level collaboration and information exchange. In this layer, each defense agent is deployed at a different node within the network and is equipped with localized observation and initial decision-making capabilities. Unlike traditional reinforcement learning settings where agents act independently, our approach incorporates a communication mechanism that enables agents to share key feature information through inter-agent messaging based on local observations. This enhances global consistency and significantly improves the flexibility and coordination of decision-making, especially in response to complex attack behaviors. The communication mechanism is essential for real-time strategy adaptation and coordination.

On top of the multi-agent communication mechanism, the system incorporates a DCGNN module. The inputs to this module include each agent’s current observation of the network topology, along with multi-dimensional state features of nodes and edges. Additionally, the input is augmented by communication information vectors received from neighboring agents. Leveraging graph convolution and neighborhood aggregation mechanisms, the DCGNN is able to extract complex node associations and infer potential attack propagation paths from the evolving network topology, thereby enabling both temporal modeling of network state changes and information sharing across multiple agents. On the output side, the DCGNN first infers probability distributions over possible actions at multiple granularities—including node-level, edge-level, and global-level. These multi-granularity action proposals are then fused within the strategy classification module, which ultimately produces the optimal discrete action decision for each agent. At the same time, the DCGNN outputs key communication vectors—refined and optimized through reinforcement learning—which are continuously exchanged among agents to share strategic intent and observation results, supporting distributed and coordinated system-wide defense. Given that the network topology itself evolves dynamically during actual attack–defense processes, DCGNN enables agents to construct more structured and adaptive feature representations, ensuring that their strategies remain closely aligned with the current environmental state.

The system’s core decision-making module is built upon a reinforcement learning framework, utilizing Proximal Policy Optimization (PPO) as the primary learning algorithm. Unlike traditional PPO methods that focus solely on optimizing individual agent returns, we incorporate game-theoretic utility functions and Bayesian belief models into the learning process by designing a joint game-theoretic loss function. This function approximates the Bayesian Nash equilibrium and integrates each agent’s long-term expected return, strategy rationality, and the overall defense performance of the system. By leveraging PPO’s stability in convergence, the model is capable of iteratively optimizing strategies over multiple interactions, thereby maximizing utility while enhancing system robustness and adaptability to evolving threats.

After strategy training is completed, the policy output and environment feedback interface is used to deploy optimized agent strategies into real or simulated network environments. The strategy output supports operations at the node, edge, and global levels, such as blocking malicious communications, restoring compromised services, or executing high-priority defense scheduling.

In summary, the JG-Defense framework establishes a foundation for strategic coordination through multi-agent communication, enhances environmental awareness through graph neural network modeling, optimizes strategic rationality and utility through a game-theoretic reinforcement learning framework, and ultimately closes the loop between defense responses and environmental feedback through a strategy deployment interface. This architecture not only is capable of managing dynamic attack–defense scenarios but also provides a practical and effective system-level solution for coordinated multi-agent defense under incomplete information conditions.

## 4. Dynamic Strategy Coordination and Optimization in Network Attack and Defense

### 4.1. Modeling Attack and Defense Environment Based on Incomplete Information

We model the attack–defense interaction as a two-coalition partially observable stochastic game with the defender coalition $B=\{B_{1},\ldots ,B_{n_{B}}\}$ and the attacker coalition $R=\{R_{1},\ldots ,R_{n_{R}}\}$. At time *t*, the two sides take joint actions $a_{B}^{t}\in \prod_{k}A_{B_{k}}$ and $a_{R}^{t}\in A_{R}=\prod_{\ell }A_{R_{\ell }}$, which induce the transition $S^{t+1}\;∼T\left(\cdot |S^{t},a_{B}^{t},a_{R}^{t}\right)$. Within this framework, the coalitions are peers at the game level, but their coordination mechanisms are heterogeneous: the defender side collaborates via multi-agent reinforcement learning, using centralized training–decentralized execution with DCGNN-based graph-structured communication; each defender agent has an independently trainable policy, and a joint game-theoretic term couples their updates at the objective level. The attacker side is multi-entity at the environment level; its coordination is expressed by a joint-action policy $\pi_{R}\left(a_{R}^{t}|\cdot \right)$ that directly defines a distribution over $A_{R}$, with each attacker sub-agent executing its corresponding component of the joint action, without independent per-agent policy optimization.

In the context of network security, network attack–defense games typically involve three types of agents defensive agents, attacking agents, and user agents, denoted as

$$B=\{B_{B},B_{R},B_{G}\}$$

Each category may consist of multiple agents. During the attack–defense process, the interaction between attackers and defenders constitutes a complex game-theoretic process. The objective of the attacker is usually to breach the defense system by various means, obtain sensitive information, or disrupt the normal operation of the system. The defender, on the other hand, is committed to constructing and maintaining effective security mechanisms to prevent penetration and sabotage by the attacker.

This type of game is highly dynamic and adversarial, with both sides continuously adjusting their strategies based on the opponent’s behavior. As attack techniques evolve, defenders must consistently optimize their defense strategies, resulting in a long-term and complex strategic confrontation. Therefore, the network attack–defense scenario can be modeled as a dynamic turn-based process.

For the *k*-th defending agent at the same round, the strategy set is

$$A_{B}=\left\{A_{B_{k}}^{t}\right\}$$

These strategy sets change dynamically with the evolution of the network scenario *S*, where the network state at round *t* is denoted as $S^{t}$.

In contrast, user agents $B_{G}$ only aim to obtain useful resources from the network and have no knowledge of the network environment. Therefore, their strategy sets remain static and do not change with the variation of the environment.

The dynamic evolution of the network environment can be simply modeled as

$$S^{t+1}=\Gamma \left(S^{t},A_{B}^{t},A_{R}^{t},A_{G}^{t}\right)$$

That is, the network state at the next round depends on the current state and the strategies taken by all agents at time *t*.

For a detailed description of the symbols used in the proposed approach, please refer to Table 1.

A distinctive feature of network attack–defense games is partial observability, where agents must make decisions under conditions of incomplete or uncertain information. Defense agents cannot fully or accurately perceive all attack behaviors. Moreover, due to the constantly changing network topology and traffic, the observation capability of the defense system is limited, making it difficult to acquire the complete state of the network in real time. At round *t*, the observation space of a defending agent *i* can be expressed as $O_{i}^{t}\in O_{i}$.

Similarly, the attacker also faces a limited observation space. The attacking agent cannot fully observe the strategies available to the defender or the structure of the current network environment. Therefore, the network attack–defense scenario can be modeled as a game of incomplete information.

Furthermore, user agents in the network exhibit stochastic behavior, and in this work, we also treat attackers as stochastic players. To construct a more realistic and effective attack–defense model, their behaviors must be considered as part of the game formulation.

### 4.2. Modeling Incomplete Information Based on Game Theory

#### 4.2.1. Derivation of the Defender’s Utility Function

In network attack–defense scenarios, the system’s utility is mainly reflected in users’ experience of the network. However, such experience does not change significantly or immediately as attacker and defender strategies vary. Attackers can disrupt services to affect normal user access, while defenders can improve general user experience through service restoration and other strategies.

Essentially, the goal of network defense is to improve user experience as much as possible and minimize the impact from attackers. The utility is inherently reflected in user experience. However, utility functions can only be defined through the strategies implemented by defenders; the function’s inputs are the environment and the defender’s strategy and cannot directly relate to users’ perceived experience. To enhance user experience, it is necessary to derive a well-constructed utility function.

Therefore, constructing a reasonable utility function that accurately captures changes under different attack–defense conditions is a crucial and challenging task. In this paper, the utility function of the defender is defined as

(1) $$\begin{array}{cc} U_{B_{j}} & =\gamma \cdot E\left[\sum_{v\in V_{critical}}\Theta \left(\sigma_{B_{j}}\left(v\right),v\right)\right]-\delta \cdot E\left[\sum_{a\in A_{B_{j}}}C_{a}\right]-\eta \cdot E\left[\sum_{g\in G}P_{g}\left(B_{j}\right)\cdot L\right] \end{array}$$

The first term, $\gamma \cdot E[\sum_{v\in V_{critical}}\Theta \left(\sigma_{B_{j}}\left(v\right),v\right)]$, reflects the expected value of implementing a specific mixed defense strategy at node vs. It is important to note that the utility value Θ is not directly measurable; it captures the complex interaction between defense strategies, network states, and resource outcomes. While Θ itself cannot be directly observed, it will be simulated within the model, learned, and adjusted based on real data, ensuring that the utility function accurately represents the resulting resource gains or losses.

The second term, $-\delta \cdot E[\sum_{a\in A_{B_{j}}}C_{a}]$, quantifies the explicit cost of each defense action *a*, ensuring that the resource expenditure of each defense strategy is clearly accounted for, thus providing a precise measurement of the direct costs associated with defense actions.

The third term, $-\eta \cdot E[\sum_{g\in G}P_{g}\left(B_{j}\right)\cdot L]$, represents the penalty for user operation failures caused by the defense action. In this case, $P_{g}\left(B_{j}\right)$ indicates the probability that a user’s operation fails due to the defensive actions of defender $B_{j}$. This penalty value *L* is derived from actual environmental feedback, ensuring that the utility function reflects the impact of defense strategies on user experience and encourages the optimization of strategies to minimize disruptions to users.

Together, these three terms form a dynamic, data-driven utility function that integrates defense strategy optimization. Through continuous model simulation and optimization, defense strategies are adjusted to enhance overall system performance.

Given the uncertainty in attacker behavior, the expected value of attacker actions is incorporated into the computation of the defender’s utility function. Specifically, the expected utility function for the defender is given by

(2) $$\begin{array}{cc} E[U_{B_{j}}] & =\gamma \cdot \sum_{v\in V_{critical}}\Theta \left(\sigma_{B_{j}}\left(v\right),v\right)-\delta \cdot \sum_{a\in A_{B_{j}}}C_{a}-\eta \cdot E\left[\sum_{g\in G}P_{g}\left(B_{j}\right)\cdot L\right] \end{array}$$

The expectation term $\sum_{g\in G}P_{g}\left(B_{j}\right)$ needs to account for the random behaviors of both user and attacker agents and their effects on the defender’s utility. $P_{g}\left(B_{j}\right)$ depends on the actions $a_{G}^{t}$ and $a_{R}^{t}$ taken by the user and attacker agents, respectively. Thus, the expectation can be expanded as

(3) $$\begin{array}{c} E\left[\sum_{g\in G}P_{g}\left(B_{j}\right)\cdot L\right]=L\cdot \sum_{g\in G}E\left[P_{g}\left(B_{j}\right)\right] \end{array}$$

Specifically,

(4) $$\begin{array}{cc} E[P_{g}\left(B_{j}\right)] & =\sum_{a_{G}^{t}\in A_{G}}\sum_{a_{R}^{t}\in A_{R}}P\left(a_{G}^{t}|S^{t}\right)\cdot P\left(a_{R}^{t}|S^{t}\right)\cdot P_{g}\left(B_{j}|a_{G}^{t},a_{R}^{t},S^{t}\right) \end{array}$$

- $P(a_{G}^{t}|S^{t})$: the probability that the user agent takes action $a_{G}^{t}$ in state $S^{t}$.
- $P(a_{R}^{t}|S^{t})$: the probability that the attacker agent takes action $a_{R}^{t}$ in state $S^{t}$.
- $P(B_{j}|a_{G}^{t},a_{R}^{t},S^{t})$: the probability that the user agent *g* fails due to the defensive action of agent $B_{j}$ given that the user and attacker take actions $a_{G}^{t}$ and $a_{R}^{t}$, respectively, in state $S^{t}$.

#### 4.2.2. Nash Equilibrium Existence Proof

Before proving the existence of Nash equilibrium, the following basic assumptions need to be clarified: We assume that the action sets of all participants (including defenders, attackers, and users) are finite. Therefore, each agent’s strategy space consists of probability distributions over its finite action set (i.e., a mixed-strategy simplex). Based on the above assumptions and in combination with the Nash equilibrium existence theorem in game theory, we further prove that the multi-agent network security game model constructed in this work necessarily possesses at least one Nash equilibrium.

The existence of Nash equilibrium relies on several fundamental mathematical conditions. According to the equilibrium existence theorems proposed by Nash [26,27] and Glicksberg [28], as long as the strategy space of each participant is a non-empty, compact, and convex set, and the utility function of each participant is continuous and quasi-concave with respect to their own strategy space, there is guaranteed to be at least one mixed-strategy Nash equilibrium in the game.

In network attack–defense games, the Nash equilibrium describes a stable combination of strategies in which each agent selects its optimal strategy, assuming that the strategies of all other agents remain unchanged. In other words, every participant makes the best choice within their own strategy space and cannot achieve a higher utility by unilaterally changing their strategy. For a defender agent $B_{j}$, if the currently adopted strategy $\sigma_{B_{j}}^{*}$ is optimal, then the utility function under the strategies of all other agents $\sigma_{B}^{*}$ satisfies the following condition:

(5) $$\begin{array}{c} U_{B_{j}}\left(\sigma_{B_{j}}^{*},\sigma_{B}^{*}\right)\geq U_{B_{j}}\left(\sigma_{B_{j}},\sigma_{B}^{*}\right)\;\forall \sigma_{B_{j}},\;B_{j}\in N_{B} \end{array}$$

This indicates that under the current strategy, the defender has already reached the best choice and has no incentive to obtain a higher utility by unilaterally deviating. Any deviation from the existing strategy cannot bring additional benefit, thus ensuring the stability of the equilibrium in the game. This property is the core of Nash equilibrium: the stability of the strategy profile.

In network attack–defense games, the strategy sets of all participating agents (defenders, attackers, and users) are represented as probability distributions.

Specifically, for a defender agent $B_{j}$, the strategy at node *v* is denoted as $\sigma_{B_{j}}\left(v\right)$, which belongs to a standard simplex:

(6) $$\Sigma_{B_{j}}\left(v\right)=\left\{\sigma |\sum_{a\in A_{B_{j}}\left(v\right)}\sigma \left(a\right)=1,\;\sigma \left(a\right)\geq 0\right\}$$

This set clearly satisfies the following properties:

- Boundedness: The strategy set forms a probability simplex, strictly confined within the closed interval [0,1], making the strategy space evidently bounded.
- Closedness: The simplex is a closed set, as every point on its boundary is included in the set itself, thereby ensuring closedness.
- Convexity: The simplex is inherently a convex set, as any linear combination of points within the set remains in the set.

Therefore, each participant’s strategy set $\Sigma_{B_{j}}\left(v\right)$ is non-empty, bounded, closed, and convex, which together ensure the compactness of the overall strategy space.

According to the defender’s utility function defined in this paper, we analyze the continuity property as follows.

The first term, $\gamma \cdot E[\sum_{v\in V_{critical}}\Theta \left(\sigma_{B_{j}}\left(v\right),v\right)]$, involves the function $\Theta (\sigma_{B_{j}}\left(v\right),v)$, which is defined by a parameterized policy network. Since the mapping from strategy probabilities to expected utility values is continuous, this term is continuous. The second term, $-\delta \cdot E[\sum_{a\in A_{B_{j}}}C_{a}]$, represents the explicit cost of defense actions and is a linear function of the strategy probabilities; thus, it is clearly continuous. The third term, $-\eta \cdot E[\sum_{g\in G}P_{g}\left(B_{j}\right)\cdot L]$, where $P_{g}\left(B_{j}\right)$ is the user failure probability and *L* is the loss, is computed as a constant or expectation over probabilities, thus constituting a linear and continuous function with respect to strategy probabilities.

Consequently, each reward and cost component is a continuous or linear function of the strategy probabilities, and the entire utility function is continuous over the strategy probability space.

To satisfy the Nash equilibrium existence theorem, each agent’s utility function must be quasi-concave with respect to its own strategy space, that is, for any fixed combination of strategies of other participants, the agent’s utility function must admit a best response within its strategy set.

In the utility function defined in this paper, each agent’s expected utility is a linear combination of expectations. With all other strategies fixed, the utility function reduces to a linear function over the agent’s own strategy set, and a linear function is both concave and convex. It is also obviously quasi-concave, since the upper-level set of a linear function is always convex.

Therefore, the defender’s utility function satisfies the quasi-concavity requirement with respect to its own strategy space given the strategies of other agents.

In summary, the defender’s strategy space is a compact and convex set, and the utility function is continuous and quasi-concave within this space. According to the Nash equilibrium existence theorem, if all participants have non-empty, compact, and convex strategy spaces, and each participant’s utility function is continuous and quasi-concave, then there exists at least one Nash equilibrium in the game. Therefore, the network attack–defense game model proposed in this paper strictly meets all conditions required by the Nash equilibrium existence theorem, ensuring that at least one Nash equilibrium solution exists within our model.

#### 4.2.3. Bayesian Nash Equilibrium Modeling

In real-world network attack–defense games, the system often involves complex multi-agent scenarios, where each agent’s decision may be influenced by incomplete and asymmetric information. In such cases, the Bayesian Nash equilibrium provides a more adaptable framework, allowing agents to choose optimal strategies probabilistically without full knowledge of others’ strategies. In network security settings, defenders must anticipate and respond to a variety of possible attack strategies, thus requiring adjustment of defensive strategies based on expected probabilities. Bayesian Nash equilibrium offers an effective solution for such decision-making under incomplete information.

Within this context, deep reinforcement learning—particularly Proximal Policy Optimization—emerges as an effective strategy optimization tool. PPO iteratively updates the policy networks of agents, enabling gradual convergence toward a globally optimal Nash equilibrium. The key strength of PPO lies in clipping the objective function to limit policy update steps, thus avoiding instability due to over-optimization. Especially in network attack–defense games, where attacker and defender strategies evolve dynamically, defenders must quickly adapt to attacker behaviors. PPO can continuously optimize defense strategies across multiple interactions, maximizing long-term cumulative rewards while ensuring stability and directional improvement. Through this adaptive adjustment, PPO drives defense strategies toward Bayesian Nash equilibrium, achieving a long-term balanced attack–defense solution.

The PPO loss function is typically defined as

(7) $$\begin{array}{cc} L_{\mathrm{PPO}} & =E\left[\min\left(r\left(\theta \right)\hat{A},\mathrm{clip}\left(r(\theta ),1-\epsilon ,1+\epsilon \right)\hat{A}\right)\right]-c_{1}L_{\mathrm{Critic}}+c_{2}H \end{array}$$

- $r\left(\theta \right)=\frac{\pi_{\theta }\left(a|s\right)}{\pi_{\mathrm{old}}\left(a|s\right)}$: policy ratio.
- $\hat{A}$: advantage estimation.
- $L_{\mathrm{Critic}}$: loss of the value network, typically mean squared error.
- H: entropy of the policy, to encourage exploration.
- $c_{1}$, $c_{2}$: weight coefficients.

This paper introduces a game-theoretic loss term, $L_{\mathrm{Game}}$, based on the utility function of agents, aiming to approach Bayesian Nash equilibrium. The game-theoretic loss is defined as the negative of total utility:

(8) $$\begin{array}{c} L_{\mathrm{Game}}=-\sum_{j=1}^{|N_{B}|}E\left[U_{B_{j}}\right] \end{array}$$

Integrating this term into the PPO loss yields

(9) $$\begin{array}{c} L_{\mathrm{Total}}=L_{\mathrm{PPO}}+\lambda L_{\mathrm{Game}}=L_{\mathrm{PPO}}-\lambda \sum_{j=1}^{|N_{B}|}E\left[U_{B_{j}}\right] \end{array}$$

- λ: weight of the game-theoretic loss in total loss.
- $|N_{B}|$: number of defender agents.

During PPO training, the goal is to maximize the expected return of the strategy while constraining the magnitude of updates. Introducing the game-theoretic loss additionally allows maximizing the expected service utility. Combined with Equation (1), the objective becomes

(10) $$\begin{array}{cc} \; & \max_{\theta }E_{\pi_{\theta }}\left[\min\left(r\left(\theta \right)\hat{A},\mathrm{clip}\left(r(\theta ),1-\epsilon ,1+\epsilon \right)\hat{A}\right)\right]+\lambda \sum_{j=1}^{|N_{B}|}E\left[U_{B_{j}}\right] \end{array}$$

During backpropagation, the gradient of the total loss with respect to parameter $\theta_{j}$ is

(11) $$\begin{array}{c} \frac{\partial L_{\mathrm{Total}}}{\partial \theta_{j}}=\frac{\partial L_{\mathrm{PPO}}}{\partial \theta_{j}}-\lambda \frac{\partial E[U_{B_{j}}]}{\partial \theta_{j}} \end{array}$$

which can be further expanded as

(12) $$\begin{array}{cc} \frac{\partial E[U_{B_{j}}]}{\partial \theta_{j}} & =\gamma \sum_{v\in V_{\mathrm{critical}}}\frac{\partial \Theta (\sigma_{B_{j}}\left(v\right),v)}{\partial \sigma_{B_{j}}\left(v\right)}\frac{\partial \sigma_{B_{j}}\left(v\right)}{\partial \theta_{j}}-\eta \cdot L\cdot \sum_{g\in G}\sum_{a_{g}^{t}\in A_{G}}\sum_{a_{r}^{t}\in A_{R}}P\left(a_{g}^{t}|S^{t}\right)P\left(a_{r}^{t}|S^{t}\right)\\ \; & \;\cdot \frac{\partial P_{g}\left(B_{j}|a_{g}^{t},a_{r}^{t},S^{t}\right)}{\partial \Theta (\sigma_{B_{j}}\left(v\right),v)}\frac{\partial \Theta (\sigma_{B_{j}}\left(v\right),v)}{\partial \sigma_{B_{j}}\left(v\right)}\frac{\partial \sigma_{B_{j}}\left(v\right)}{\partial \theta_{j}} \end{array}$$

Thus, the final strategy parameter update rule is

(13) $$\begin{array}{c} \theta_{j}\leftarrow \theta_{j}-\eta \left(\frac{\partial L_{\mathrm{PPO}}}{\partial \theta_{j}}-\lambda \frac{\partial E[U_{B_{j}}]}{\partial \theta_{j}}\right) \end{array}$$

Therefore, through backpropagation, both the PPO loss and the game-theoretic loss can be optimized simultaneously, enabling defender strategies to maximize long-term returns while minimizing negative effects caused by stochastic attacker behavior and user agent randomness.

### 4.3. Bayesian Belief Update Modeling

In dynamic network attack–defense scenarios characterized by incomplete information, the core function of the belief model lies in dynamically adjusting the estimation of attacker behavior based on observed data. For defenders, the state of the attacker is not directly observable and must instead be inferred from observations $O_{B_{j}}$. Therefore, a belief model is required to estimate the probability distribution over the attacker’s actions. In this paper, the belief model is based on Bayesian theory to update the probability distribution of attacker actions. By dynamically integrating observations and prior information, the model optimizes the estimation of attacker behavior to support further defensive decision-making. Let $O_{B_{j}}^{t}$ be the observation information of defender agent $B_{j}$ at time *t*. The belief about attacker action $a_{R}^{t}$, where $a_{R}^{t}\in A_{R}$, is represented as $P(a_{R}^{i}|O_{B_{j}}^{t})$.

To dynamically adjust judgments of attacker actions, the Bayesian update mechanism dictates that when agent $B_{j}$ takes action $A_{t}^{B}$ at time *t* and receives new observation $O_{B_{j}}^{t+1}$, its belief about attacker action $a_{R}^{t}$ is updated. According to Bayes’ theorem, the updated belief is

(14) $$\begin{array}{cc} P(a_{R}^{t}|O_{B_{j}}^{t+1}) & =\frac{P\left(O_{B_{j}}^{t+1}|a_{R}^{t},O_{B_{j}}^{t}\right)\cdot P\left(a_{R}^{t}|O_{B_{j}}^{t}\right)}{P(O_{B_{j}}^{t+1}|O_{B_{j}}^{t})} \end{array}$$

- $P(O_{B_{j}}^{t+1}|a_{R}^{t},O_{B_{j}}^{t})$: the conditional probability of receiving new observation $O_{B_{j}}^{t+1}$ given that the attacker took action $a_{R}^{t}$ and the previous observation was $O_{B_{j}}^{t}$.
- $P(O_{B_{j}}^{t+1}|O_{B_{j}}^{t})$: the marginal probability of observing $O_{B_{j}}^{t+1}$, which can be computed via the law of total probability:
  $$\begin{array}{c} \begin{array}{cc} P(O_{B_{j}}^{t+1}|O_{B_{j}}^{t}) & =\sum_{a_{R}^{t}\in A_{R}}P\left(O_{B_{j}}^{t+1}|a_{R}^{t},O_{B_{j}}^{t}\right)\cdot P\left(a_{R}^{t}|O_{B_{j}}^{t}\right) \end{array} \end{array}$$

The core of Bayesian belief updating lies in dynamically adjusting the distribution over attacker behavior and incorporating it into the utility function to optimize defender strategies. In network security scenarios, this mechanism provides defender agents with timely and reliable bases for adjusting strategies. Especially when facing dynamically evolving attack–defense conditions, belief updates significantly enhance system utility and achieve effective strategic balance.

The expected utility for defender agent $B_{j}$ needs to be computed based on updated beliefs. By combining Equations (2)–(4), we obtain

(15) $$\begin{array}{cc} E[U_{B_{j}}] & =\gamma \cdot \sum_{v\in V_{\mathrm{critical}}}\Theta \left(\sigma_{B_{j}}\left(v\right),v\right)-\delta \cdot \sum_{a\in A_{B_{j}}}C_{a}\\ \; & \;-\eta \cdot L\cdot \sum_{g\in G}\sum_{a_{G}^{t},a_{R}^{t}}P\left(a_{G}^{t}|S^{t}\right)\cdot P\left(a_{R}^{t}|O_{B_{j}}^{t}\right)P_{g}\left(B_{j}|a_{G}^{t},a_{R}^{t},S^{t}\right) \end{array}$$

Here, $P(a_{R}^{t}|O_{B_{j}}^{t})$ reflects the belief of defender agent $B_{j}$ about attacker behavior given its observed information.

## 5. Multi-Agent Joint Game-Theoretic Decision Making

### 5.1. Multi-Agent Cooperative Optimization

In network attack–defense settings, a defense system’s performance typically hinges on the coordination and cooperation among multiple defensive agents. These agents must not only optimize their own policies independently but also collaborate at the system level to ensure global policy consistency. In multi-agent environments, a key challenge is how to optimize each agent’s policy so that, when facing complex attack behaviors, they can act in concert and realize a system-level advantage.

To this end, the policy parameters of all defender agents are jointly optimized. Let the policy parameters of all defenders be

$$\begin{array}{c} \theta =\{\theta_{B_{1}},\theta_{B_{2}},\ldots ,\theta_{B_{m}}\}. \end{array}$$

The joint optimization objective is defined as

(16) $$\begin{array}{c} L_{\mathrm{Total}}=L_{\mathrm{PPO}}+\lambda L_{\mathrm{Game}}=L_{\mathrm{PPO}}-\lambda \sum_{j=1}^{|N_{B}|}E\left[U_{B_{j}}\right] \end{array}$$

where each component of the policy optimization is given by

(17) $$\begin{array}{cc} L_{\mathrm{PPO}}^{B_{j}} & =E\left[\min\left(r\left(\theta_{j}\right){\hat{A}}_{j},\mathrm{clip}\left(r(\theta_{j}),1-\epsilon ,1+\epsilon \right){\hat{A}}_{j}\right)\right]\\ \; & \;-c_{1}L_{\mathrm{Critic}}^{B_{j}}+c_{2}H^{B_{j}}, \end{array}$$

(18) $$\begin{array}{cc} L_{\mathrm{Game}}^{B_{j}} & =-E[U_{B_{j}}]. \end{array}$$

Under the two-coalition (defender–attacker) formulation, we align the team’s instantaneous reward $r_{B}$ with the sum of individual utilities and write the training objective as a best response to a fixed or aggregate attacker distribution $\pi_{R}$:

(19) $$\begin{array}{c} \max_{\theta_{B}}\;E_{\pi_{B}^{\theta_{B}},\;\pi_{R}}\left[\sum_{t}\gamma^{t}\;r_{B}\left(S^{t},a_{B}^{t},a_{R}^{t}\right)\right]. \end{array}$$

(20) $$\begin{array}{c} r_{B}\left(S^{t},a_{B}^{t},a_{R}^{t}\right)\;≜\;\sum_{j=1}^{|N_{B}|}U_{B_{j}}\left(S^{t},a_{B}^{t},a_{R}^{t}\right). \end{array}$$

This objective is consistent with the optimization direction of (16): by introducing the joint game term, $L_{\mathrm{Total}}$ effectively encourages policies that maximize long-horizon team return while discouraging individually irrational behavior. For ease of reproduction and to keep the focus on the method’s main axes, our implementation adopts a fixed or aggregate $\pi_{R}$. If the attacker side is also made learnable, $\pi_{R}^{\phi_{R}}$, we obtain the standard min–max extension:

(21) $$\begin{array}{c} \max_{\theta_{B}}\;\min_{\phi_{R}}\;E_{\pi_{B}^{\theta_{B}},\;\pi_{R}^{\phi_{R}}}\left[\sum_{t}\gamma^{t}\;r_{B}\left(S^{t},a_{B}^{t},a_{R}^{t}\right)\right]. \end{array}$$

Through joint optimization of policy objectives and regularization constraints, the system can improve performance while maintaining the robustness and consistency of the policies. The gradient of the utility function can be written as

(22) $$\begin{array}{cc} \frac{\partial E[U_{B_{j}}]}{\partial \theta_{j}} & =\gamma \cdot \sum_{v\in V_{critical}}\frac{\partial \Theta (\sigma_{B_{j}}\left(v\right),v)}{\partial \sigma_{B_{j}}\left(v\right)}\frac{\partial \sigma_{B_{j}}\left(v\right)}{\partial \theta_{j}}-\eta \cdot L\cdot \sum_{g\in G}\sum_{a_{g}^{t}\in A_{g}}\sum_{a_{r}^{t}\in A_{r}}P\left(a_{g}^{t}|S^{t}\right)P\left(a_{r}^{t}|O^{t}\right)\\ \; & \;\cdot \frac{\partial P_{g_{j}}\left(B_{j}|a_{g}^{t},a_{r}^{t},S^{t}\right)}{\partial \Theta (\sigma_{B_{j}}\left(v\right),v)}\frac{\partial \Theta (\sigma_{B_{j}}\left(v\right),v)}{\partial \sigma_{B_{j}}\left(v\right)}\frac{\partial \sigma_{B_{j}}\left(v\right)}{\partial \theta_{j}} \end{array}$$

This shows that during the optimization process, each agent’s independent policy performance is considered, and so are the collaborative interactions between agents and the balance of interests among all parties in the game. Through these mechanisms, the entire system can achieve long-term stable and optimal defense strategies.

### 5.2. DCGNN: A Multi-Agent Joint Communication Game-Theoretic Decision Network Based on Graph Neural Networks

**Figure 2:**
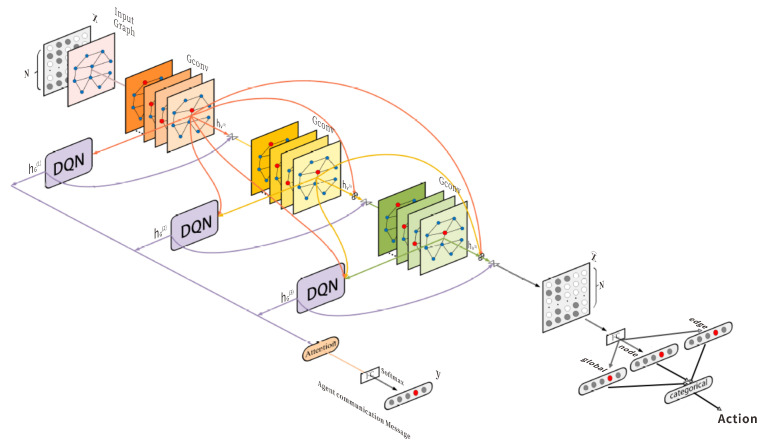
Architecture of the DCGNN.

This study proposes a multi-agent joint game-theoretic defense model based on graph neural networks, named DCGNN. The overall architecture is illustrated in Figure 2. With network topology as input, the model first employs graph convolutional networks to extract initial structural features. These features are then fed into Deep Q-Networks to generate preliminary action-value estimates for each node. To further enhance representational capability, the model adopts a layer-wise iterative mechanism, stacking three sets of GCN and DQN modules. Each DQN receives the graph embedding output from the preceding GCN layer as input, progressively refining both local and global situational modeling of each node.

In the final layer, the system outputs an intermediate feature vector, which is passed to a Multi-Layer Perceptron to produce an eight-dimensional communication message vector. This vector is used to share information among multiple defense agents. Simultaneously, node-level, edge-level, and global-level action probability distributions are generated by the final DQN and integrated into a policy classification module that ultimately produces discrete action decisions for the agents.

Throughout the training process, the model employs the Proximal Policy Optimization reinforcement learning algorithm. The actor network is responsible for generating both policy outputs and communication information, while the critic network evaluates the current state’s value. The actor network supports strategy outputs at the node, edge, and global levels, enabling the model to produce fine-grained defense actions according to different granularities of network state. Through its communication mechanism, critical information is shared among agents to enhance coordination. The critic network outputs a scalar representing the overall value of the current strategy, serving as feedback to help the actor optimize its outputs. This architectural design enables continuous policy improvement and stable game performance over long-term tasks.

The core idea of the model is to treat the network environment as a graph structure. Nodes (such as hosts, servers, routers) and edges (such as connection links) naturally form a graph, where nodes represent entities and edges represent their interactions. GNNs, as deep learning models specialized for graph-structured data, can capture both local structure and global dependencies through iterative feature passing and aggregation among nodes. This allows efficient mining of latent threat patterns and attack propagation paths in the network. Within the multi-agent game framework proposed here, GNNs are used not only for feature extraction but also to serve as the foundation for the communication mechanism, enabling agents to share local observations and form a consistent global strategy.

In practical attack–defense scenarios, attacker behavior often manifests as anomalous activity propagating from a single node to neighboring ones. This pattern is well-suited to modeling and detection via GNNs. Through graph-based propagation mechanisms, agents can infer potential threats not only from their own states but also from the states of neighboring nodes, enabling proactive responses to unfolding attacks. Furthermore, the GNN layers extract strategic information at multiple granularities: node-level strategies such as node recovery or service shutdown, edge-level strategies such as link disconnection or path blocking, and global-level strategies encompassing overall scheduling and resource allocation. This multi-granularity abstraction tightly integrates with the nested graph structure, ensuring the flexibility and consistency of the strategy output with the environment.

In conclusion, the proposed DCGNN model, driven by graph neural networks and designed for multi-agent joint game-theoretic defense, effectively combines three core mechanisms: structural modeling, policy optimization, and information transmission. The embedded GNN module not only enhances dynamic environment modeling but also enables precise and structured communication. The resulting policies exhibit both global optimality and inter-agent coordination, providing a robust end-to-end decision-making solution for complex and dynamic network defense scenarios.

## 6. Experiments

### 6.1. Experimental Scenario and Design

This study aims to experimentally investigate the following core research questions:

- RQ1: Can the proposed JG-Defense method, by introducing a joint game-theoretic loss function, comprehensively optimize multi-agent strategic utility in incomplete information games, thereby achieving higher strategy coordination and system robustness in dynamic network defense environments?
- RQ2: Does the proposed DCGNN effectively extract critical topological structures and evolving attack patterns in dynamic network environments? Can its multi-layer graph convolutional architecture enhance the defense agents’ perception and response to complex environmental changes?
- RQ3: Does the multi-agent communication mechanism introduced in the DCGNN enable efficient information sharing and contextual reasoning? Can it strengthen strategy coordination among agents, thereby enhancing the effectiveness and robustness of the joint game-theoretic model in practical attack–defense scenarios?

The software and hardware environment used in the experiments is shown in Table 2.

**Figure 3:**
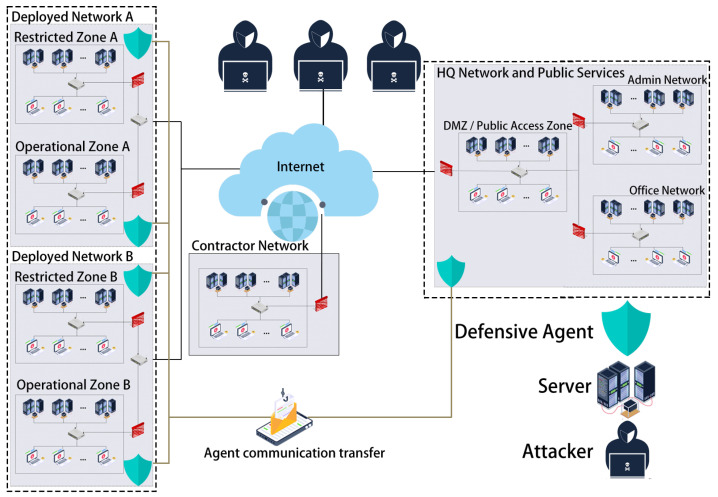
Network attack–defense scenario.

To verify the above research questions, the experiment uses the Cage4 scenario [29] as the experimental platform, as illustrated in Figure 3. Cage4 consists of four subnetworks: the Headquarters Network, the Contractor Network, and two Deployed Networks. These networks are interconnected via the Internet. Each Deployed Network is divided into a Restricted Zone and an Operational Zone, where traffic must pass through the Restricted Zone to enter the Operational Zone. The Headquarters Network includes a Public Access Area, an Administrative Zone, and an Office Area. Each zone contains 1–6 servers and 3–10 hosts, with each host/server running at least one and up to five service programs.

The evaluation criterion is based on user agent experience. When a user agent requests access to a node and fails, a penalty is applied. The task process is divided into three phases: Phase 1 is a routine operation phase where all regions have equal task importance. Phase 2 is split into 2A and 2B, corresponding to task activation in Deployed Networks A and B, respectively. In 2A, Operational Zone A becomes the core area, while in 2B, the focus shifts to Deployed Network B. User agents initiate local or cross-zone service requests, and if these requests fail due to host unavailability, service disruption, or network policy restrictions, penalties are incurred. The specific penalty standards are listed in Table 3.

To quantitatively map Equations (1)–(3) to the actual experimental environment, when a user agent accesses a service at different stages, if the target node or service is not started or becomes unreachable due to erroneous blocking by a defensive strategy, a corresponding penalty score *L* is triggered. All penalty values are directly taken from Table 3 and serve as the experimental values of the loss term *L* in the formulas; during training, samples are drawn uniformly over time. Meanwhile, the node expected reward function $\Theta (\sigma_{B_{j}}\left(v,v\right))$ is learned end-to-end via the DCGNN.

### 6.2. The Role of the JG-Defense Method in Optimizing Defense Strategies Through Joint Game Theory

**Figure 4:**
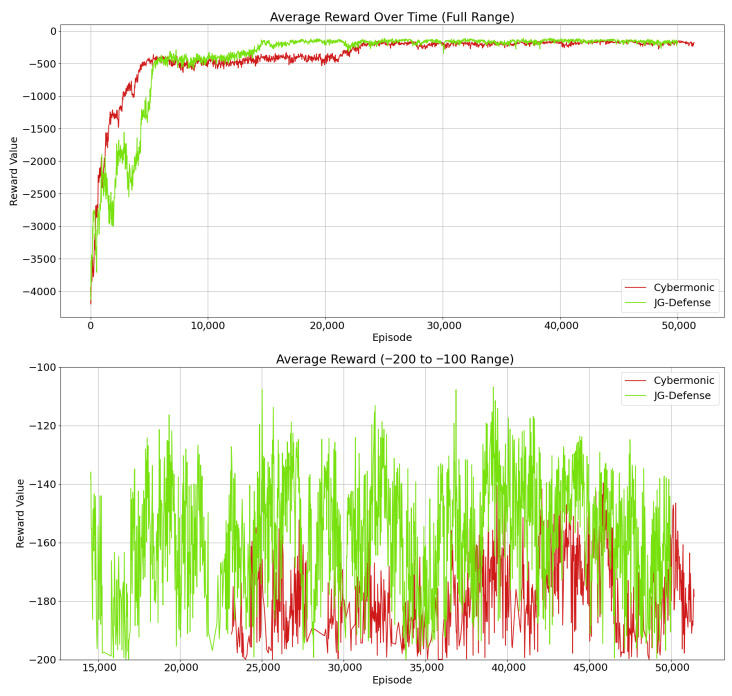
Performance evaluation of joint game in defense strategy optimization.

To address RQ1—i.e., whether the proposed JG-Defense method can optimize multi-agent strategy utility in the game process through the joint game loss function, thereby enhancing the overall robustness and coordination of the defense system—we designed this comparative experiment. The experiment compares the JG-Defense method with Cybermonic [30] to verify the contribution of the joint game mechanism in strategy coordination and utility optimization. The results show that the JG-Defense method achieves a test reward of −145.35 after training convergence, while the Cybermonic method scores −170.32, demonstrating a significant improvement in defense performance for the former. Detailed results are shown in Figure 4.

From the results, it is evident that the introduction of the joint game loss function significantly enhances the collaborative strategies among defensive agents. Although the score improves slowly during the early training phase due to repeated strategy adjustments, the collaboration gradually improves, and the effectiveness of defense strategies becomes increasingly prominent. The upper part of the graph shows the variation in reward across the full training span. Notably, models with the joint game loss quickly widen the performance gap, especially in high-reward scenarios, indicating notable gains in defense performance.

The introduction of a joint game loss enables the model to dynamically adjust its strategy in response to the environment. Defensive agents not only consider their own utility but also others’ behavior, effectively avoiding local optima. In the later stages of training, the defense system becomes more stable and reaches higher scores, validating the effectiveness of the joint game loss in optimizing defense strategies in multi-agent systems.

### 6.3. Feature Extraction Capability of DCGNN in Dynamic Network Environments

**Figure 5:**
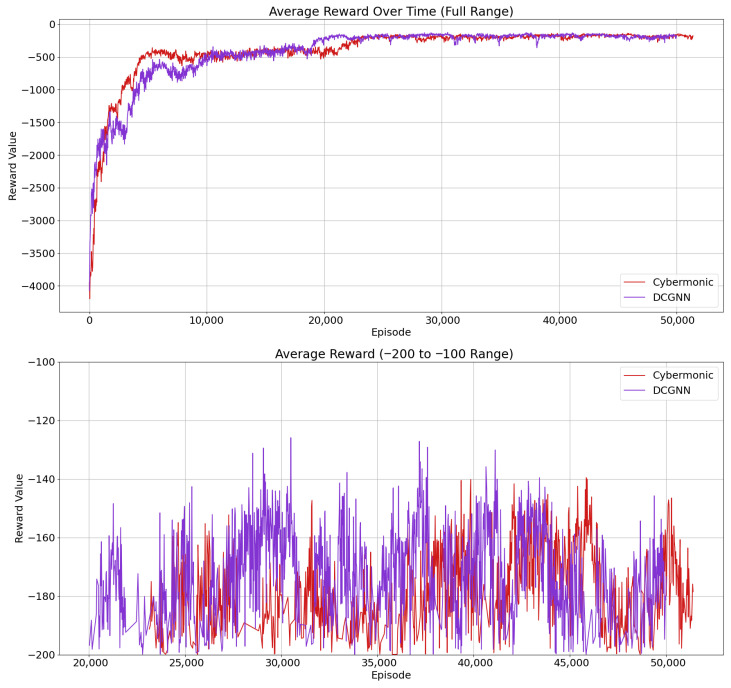
Feature extraction capability of GNN in network environments.

To answer RQ2—namely, to evaluate whether the proposed DCGNN is capable of accurately extracting critical topological structures and attack evolution patterns in dynamic network environments and whether it can effectively enhance the perception and response performance of defensive agents to complex environmental changes through multi-layer graph convolution mechanisms—we designed a set of comparative experiments. This experiment focuses on analyzing the contribution of the DCGNN model itself to optimizing defense strategies in the absence of a game-theoretic mechanism. Specifically, two models were compared: one employing the DCGNN model, and the other using Cybermonic’s network model. The comparison results show that the DCGNN model achieved a test reward value of −150.20 after convergence, whereas the Cybermonic model reached −170.32 under the same conditions. The detailed trend of experimental scores is shown in Figure 5.

The experimental results indicate that the DCGNN model showed only minor differences compared to the Cybermonic model throughout the training process. Although the Cybermonic model had a slight advantage in the early stages of training, the DCGNN model surpassed it from the mid-stage onward. As shown in the upper section of the figure, the reward values of the DCGNN model remained consistently high throughout the training process. Especially in the later stages, the DCGNN’s scores steadily increased and exceeded those of the Cybermonic model, demonstrating its defensive performance.

Compared with the Cybermonic model, the DCGNN exhibited superior capabilities in handling the dynamic topology of networks and was able to update node status information in the network environment in real time. The final experimental results confirm the DCGNN’s critical role in optimizing defense strategies in dynamic network environments. In particular, the DCGNN demonstrated strong potential in extracting complex network features and adapting to evolving attacker behavior.

### 6.4. Impact of Inter-Agent Communication in DCGNN on Defense Performance

**Figure 6:**
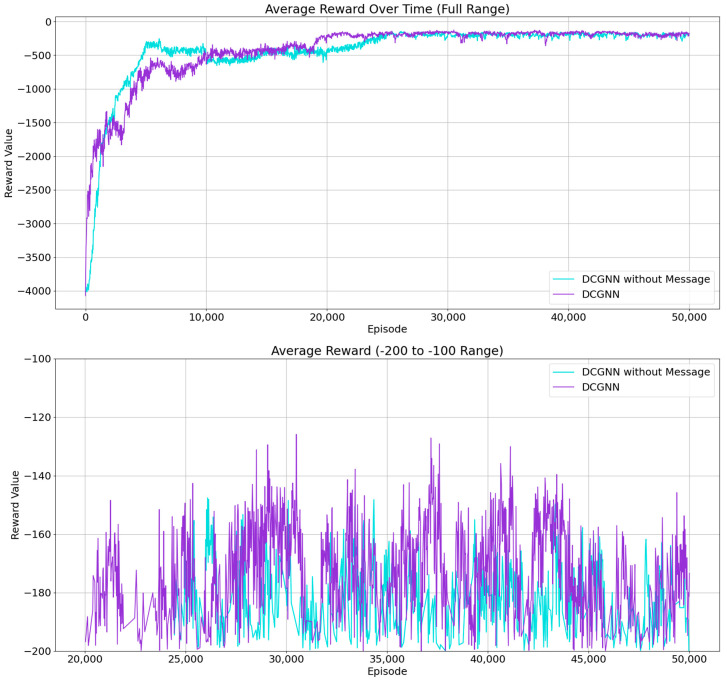
Impact of multi-agent communication mechanism on defense strategies.

To answer RQ3—i.e., whether the multi-agent communication mechanism in the DCGNN enables efficient information sharing and contextual reasoning, enhancing coordination among strategies and improving the effectiveness and robustness of the defense model in practical attack–defense scenarios—we designed this comparative experiment. Two setups were used: one with communication enabled (agents share local observations), and one without communication (agents make independent decisions with no information exchange). Results show that the communication-enabled DCGNN achieves a reward of −150.20 after convergence, while the non-communicative DCGNN scores −169.67. Detailed score trends are shown in Figure 6.

The communication-enabled DCGNN model significantly outperforms its counterpart in terms of strategy coordination and stability. The figures show consistently high and stable reward values throughout training for the communicative model. In contrast, although the non-communicative model improves slightly, it remains inferior. The communicative model demonstrates stronger stability and higher scores in reward improvements.

When communication is enabled, agents can share local observations in real time, allowing more coordinated defense decisions, reducing conflicts and redundancy. This is especially beneficial in complex attack scenarios where attackers are multiple and behavior is dynamic. Agents using communication can respond more flexibly and precisely. Without communication, coordination is poor, leading to suboptimal system performance, especially when rapid attacker changes occur, lowering response speed and adaptability.

## 7. Conclusions

The network attack–defense scenario represents a typical dynamic game process in which both attackers and defenders continuously adjust their strategies, resulting in complex and highly adversarial interactions. In this process, the strategy space of both parties is finite and expressed in the form of mixed strategies. Through subtle strategy adjustments, these strategies can transition smoothly within the state space, thus theoretically ensuring the existence of at least one Nash equilibrium. Based on this, this paper introduces a novel loss function grounded in game-theoretic principles, designed for multi-agent joint network attack–defense games. This function aids defensive agents in more effectively optimizing their strategies and maximizing their long-term rewards.

Furthermore, to better characterize the multi-level nature of network attack–defense scenarios, this paper proposes the DCGNN, which models the strategy space at three hierarchical levels: node-level strategies, edge-level strategies, and global-level strategies. This hierarchical strategy modeling fully leverages the network topology and the dynamic nature of attack–defense interactions, enabling the model to generate optimal strategies at multiple levels that are suitable for the current network state. Experimental results demonstrate that the proposed method achieves significant improvements in both strategy precision and dynamic adaptability.

Looking ahead, we will relax the simplifying assumption on the attacker and instantiate a learning, multi-agent adversary. Specifically, we will undertake the following: (i) endow each attacker with an independently optimizable policy and compare three training configurations—parameter sharing, CTDE, and fully decentralized; (ii) study three cooperation mechanisms—joint action, shared value, and message communication—and quantify their impact on equilibrium selection and robustness; and (iii) adopt stronger adversarial training protocols (alternating updates, fictitious self-play with opponent pools, and domain randomization) to stress-test convergence and generalization.

This paper presents an innovative solution to the multi-agent joint network defense problem by integrating game-theoretic foundations with the modeling capabilities of graph neural networks. Future research will focus on enhancing the realism and complexity of attack–defense modeling, exploring more efficient and intelligent strategy optimization methods within the frameworks of incomplete-information games and multi-agent deep reinforcement learning.

## Figures and Tables

**Table 1:** Symbols used in the proposed approach.

Symbol	Description
$B=\{B_{B},B_{R},B_{G}\}$	Set of agents: defenders ($B_{B}$), attackers ($B_{R}$), and users ($B_{G}$)
$A_{B_{k}}^{t}$	Strategy set of defender agent *k* at time *t*
$A_{R_{k}}^{t}$	Strategy set of attacker agent *k* at time *t*
$S^{t}$	Network state (scenario) at round *t*
$S^{t+1}=\Gamma \left(S^{t},A_{B}^{t},A_{R}^{t},A_{G}^{t}\right)$	Network transition function
$O_{i}^{t}\in O_{i}$	Observation space of agent *i* at time *t*
γ	Weight for service continuity
δ	Weight for defense cost
η	Penalty weight for user failure due to defense
$C_{a}$	Cost of executing defense action *a*
*L*	Loss from failed user operation due to defense
$\sigma_{B_{j}}\left(v\right)$	Mixed defense strategy of $B_{j}$ at node *v*
$\Theta (\sigma_{B_{j}}\left(v\right),v)$	Utility of defense strategy at node *v*
$P_{g}\left(B_{j}\right)$	Probability that defense of $B_{j}$ causes user *g* to fail
$P(a_{G}^{t}|S^{t})$	Probability user agent takes action $a_{G}^{t}$ in state $S^{t}$
$P(a_{R}^{t}|S^{t})$	Probability attacker takes action $a_{R}^{t}$ in state $S^{t}$
$P_{g}\left(B_{j}|a_{G}^{t},a_{R}^{t},S^{t}\right)$	Conditional probability of user *g* failure under actions
$L_{\mathrm{PPO}}$	PPO loss function
$L_{\mathrm{Critic}}$	Value network loss
H	Policy entropy for exploration
r(θ)	Policy ratio $\pi_{\theta }\left(a|s\right)/\pi_{\mathrm{old}}\left(a|s\right)$
$\hat{A}$	Advantage estimate
$L_{\mathrm{Game}}$	Game-theoretic loss (negative utility sum)
$R=\{R_{1},\ldots ,R_{n_{R}}\}$	Attacker coalition
$A_{R}=\prod_{\ell }A_{R_{\ell }}$	Joint action space
$a_{R}^{t}\in A_{R}$	Joint attacker action at *t*
$L_{\mathrm{Total}}$	Total loss with game-theoretic term
$\theta_{j}$	Strategy parameter of defender agent $B_{j}$
$V_{\mathrm{critical}}$	Set of critical nodes
$\Sigma_{B_{j}}\left(v\right)$	Strategy space of defender $B_{j}$ at node *v*
$P(a_{R}^{t}|O_{B_{j}}^{t})$	Belief over attacker actions based on observation
$O_{B_{j}}^{t}$	Observation of defender $B_{j}$ at time *t*
λ	Weight of game-theoretic loss in total loss

**Table 2:** Experimental environment.

Component	Specification
OS	Ubuntu 20.04
CPU	16 vCPU Intel(R) Xeon(R) Platinum 8474C
RAM	80 GB
GPU	A4000
Anaconda	4.10.3
Keras	2.6
Python	3.8

**Table 3:** Penalty scores for green agent failures.

Action	Phase 1	Phase 2	Phase 3
HQ Network Local Work Fails	−1	−1	−1
HQ Network Access Service Fails	−1	−1	−1
HQ Network Red Impact/Access	−3	−3	−3
Contractor Network Local Work Fails	0	0	0
Contractor Network Access Service Fails	−5	0	0
Contractor Network Red Impact/Access	−5	0	0
Restricted Zone A Local Work Fails	−1	−2	−1
Restricted Zone A Access Service Fails	−3	−1	−3
Restricted Zone A Red Impact/Access	−1	−3	−3
Operational Zone A Local Work Fails	−1	−10	−1
Operational Zone A Access Service Fails	−1	0	−1
Operational Zone A Red Impact/Access	−1	−10	−1
Restricted Zone B Local Work Fails	−1	−1	−2
Restricted Zone B Access Service Fails	−3	−1	−1
Restricted Zone B Red Impact/Access	−1	−1	−3
Operational Zone B Local Work Fails	−1	−1	−10
Operational Zone B Access Service Fails	−1	−1	−1
Operational Zone B Red Impact/Access	−1	−1	−10
Internet Local Work Fails	0	0	0
Internet Access Service Fails	0	0	0
Internet Red Impact/Access	0	0	0

## Data Availability

The experimental scenarios used in this study can be accessed at the following GitHub repository: https://github.com/cage-challenge/cage-challenge-4 (accessed on 4 June 2024).
